# Therapeutic Value of *Ginkgo biloba* Extract EGb 761^®^ in an Animal Model (*Meriones unguiculatus*) for Noise Trauma Induced Hearing Loss and Tinnitus

**DOI:** 10.1371/journal.pone.0157574

**Published:** 2016-06-17

**Authors:** Patrick Krauss, Konstantin Tziridis, Stefanie Buerbank, Achim Schilling, Holger Schulze

**Affiliations:** Experimental Otolaryngology, Friedrich-Alexander Universität Erlangen-Nürnberg, Waldstrasse 1, 91054, Erlangen, Germany; The Research Center of Neurobiology-Neurophysiology of Marseille, FRANCE

## Abstract

Noise induced hearing loss (NIHL) is a common disease in modern societies and may lead to maladaptations within the auditory system that finally result in subjective tinnitus. Available therapies may only alleviate the symptoms rather than restore normal hearing. In a previous study we demonstrated that the prophylactic application of *Ginkgo biloba* extract EGb 761^®^ significantly reduces NIHL and tinnitus development in our Mongolian gerbil (*Meriones unguiculatus*) animal model. Here, we tested whether the application of EGb 761^®^ has beneficial effects after the formation of permanent NIHL and tinnitus. To this end we monitored the therapeutic effects of EGb 761^®^ on noise trauma-induced changes in signal processing within the auditory system of our animal model by behavioral (acoustic startle response, ASR) and electrophysiological approaches (auditory brainstem responses, ABR). We found that–in contrast to vehicle–three weeks of daily oral EGb 761^®^ treatment (100 mg/kg body weight) led to a restoration of hearing thresholds back to pre-trauma conditions. In addition, all 9 animals that displayed behavioral signs of subjective tinnitus showed improvement, with 7 of them showing complete relief of tinnitus symptoms during the time of EGb 761^®^ treatment. After discontinuation of EGb 761^®^ treatment, tinnitus related behavior reappeared in all but one of these animals while auditory thresholds remained restored. A detailed analysis of ABR waves revealed that EGb 761^®^ treatment did not simply change auditory processing back to pre-trauma conditions, but led to subtle changes of ABR wave amplitude and latency at different levels of the auditory pathway, with an overall increase of response to low stimulus intensities and a decrease at high intensities. The functional relevance of these changes may be the observed improvement of hearing thresholds while at the same time suppression of responses to high stimulus intensities may point to a global inhibitory mechanism that counteracts tinnitus.

## Introduction

The steadily increasing levels of noise exposure within our working environments and during leisure time activities (for review see [1]) results in an increasing number of people suffering from noise-induced hearing loss (NIHL). As hearing loss is linked to the development of a number of secondary diseases, like hyperacusis (for review see [2]), tinnitus [3, 4] or depression due to social isolation [5] the problem of NIHL will have an immensely growing impact on our health systems. Between 5% and 15% of the general population report to be affected by tinnitus and around 1% state that their quality of life is considerably impaired by the condition [6].

Over the last few years a large number of substances have been tested both in animal and human studies in search for a drug that is able to restore hearing function after NIHL (for review see [7]). Based on their physiological mechanisms, a number of substance classes may be distinguished. Among these, antioxidants that reduce oxidative stress by elimination of reactive oxygen species (ROS) [8], glucocorticoids and substances that improve cochlear blood flow (for review see [9]), activate inhibitory transmitter systems [10] or block apoptosis pathways in hair cells were most successfully employed (for review see [11]).

A substance that has been discussed as a potential therapeutic in the context of NIHL and tinnitus is the *Ginkgo biloba* extract EGb 761^®^, which contains about 250 different compounds deploying a number of different mechanisms. Well-documented in animal and also human studies are the protection of neuronal mitochondrial ATP synthesis in the presence of oxidative stress [12, 13], the protection of erythrocyte membranes against oxidative damage, which results in reduced blood viscosity and improved blood flow [14, 15], and neuroprotection through antiapoptotic properties [16–20]. In addition, the extract displays a number of effects within the central nervous system that may enhance neuronal plasticity and neurotransmitter levels, and therefore might be beneficial in tinnitus. These include increased extracellular dopamine levels in the prefrontal cortex [21] which may reduce depressive behavior by partial inhibition of the norepinephrine transporter [22], or neuroneogenesis of hippocampal neurons [23] which could both lead to cognition increasing effects. In clinical trials, EGb 761^®^ was well tolerated and showed a favorable safety profile [24]. Therefore, EGb 761^®^ seems to be a promising candidate substance for the treatment of NIHL.

It has been shown previously that EGb 761^®^ is able to protect against NIHL in guinea pigs [25, 26]. Stange and co-workers demonstrated that animals which were treated with EGb 761^®^ before exposing them to different types of noise trauma exhibited a smaller reduction in auditory nerve compound action potentials (CAP) than untreated controls.

Recently we were able to substantiate the results of Stange and co-workers in our animal model, the Mongolian gerbil (*Meriones unguiculatus*). We showed that the prophylactic application of EGb 761^®^ significantly reduces NIHL, and in addition we could show that also the subsequent development of tinnitus was significantly reduced in treated animals [27]. Nevertheless, although our finding of a prophylactic effect of EGb 761^®^ against NIHL and tinnitus is promising, its practical potential is in principle limited to a small group of individuals, namely those exposed to severe but temporally unpredictable noise exposure, e.g. to soldiers or construction workers. Several other groups and companies work on a pharmacotherapy against tinnitus in a chronic state (for review see [28]). Among the tested substances also EGb 761^®^ is a promising candidate for the development of a treatment against this phantom percept.

In the present study we therefore investigated if the application of EGb 761^®^ after the formation of permanent NIHL and tinnitus still has beneficial effects. To this end we monitored the effects of EGb 761^®^ on noise trauma-induced changes in signal processing within the auditory system of our animal model by behavioral (acoustic startle response, ASR) and electrophysiological approaches (auditory brainstem responses, ABR.)

## Materials and Methods

### Ethics statement and animals

Mongolian gerbils (*Meriones unguiculatus*) were housed in standard animal racks (Bio A.S. Vent Light, Ehret Labor- und Pharmatechnik, Emmendingen, Germany) in groups of 2 to 3 animals per cage with free access to water and food at 20 to 24°C room temperature under 12/12 h dark/light cycle. The use and care of animals was approved by the state of Bavaria (Regierungspräsidium Mittelfranken, Ansbach, Germany; AZ: 54–2532.1-02/13).

A total of 20 male gerbils aged ten to twelve weeks purchased from Charles River Laboratories Inc. (Sulzfeld, Germany) were used in this study. All methods as well as the animal model (cf. Discussion) have been described previously [27, 29, 30] but will be explained here briefly.

### Time regime and treatment with EGb 761^®^

**Fig 1:**
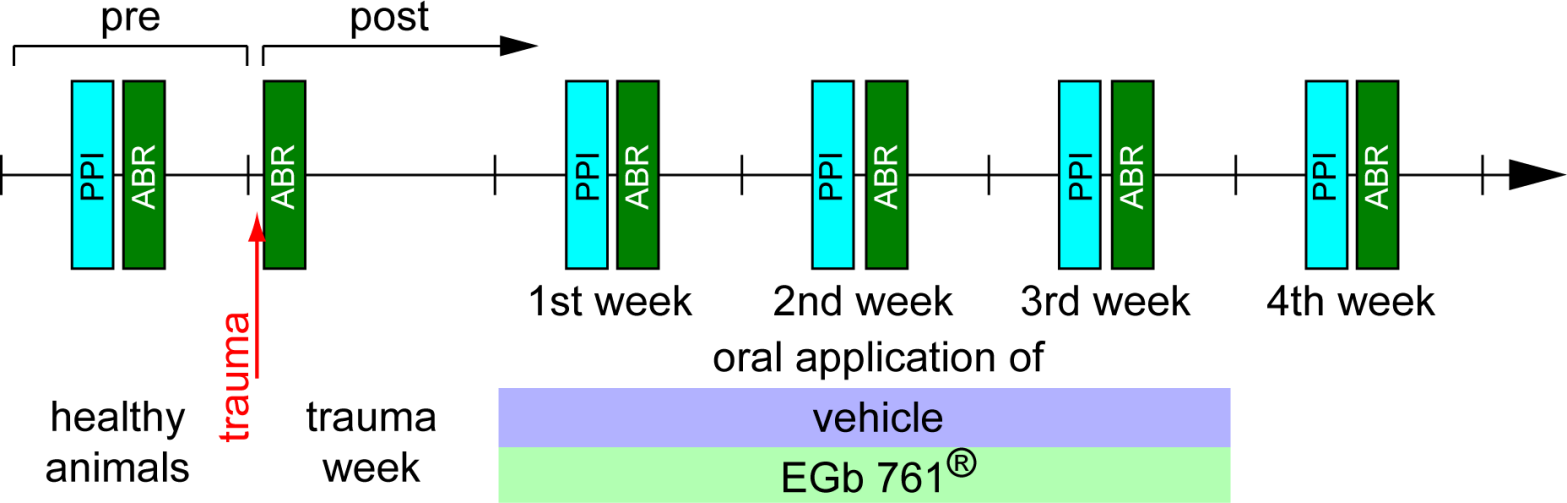
Experimental protocol. PPI of the ASR was always measured ahead of ABR measurements. The trauma consisted of a 2 kHz pure tone, presented for 75 min at 115 dB SPL. Animals were either treated with vehicle (2% Agar in water) or EGb 761^®^ (100mg/kg in 2% Agar in water).

All 20 animals were handled before the beginning of the experiments and accustomed to the setup environment to minimize stress. An overview of the experimental protocol is depicted in Fig 1. First, healthy animals (pre-trauma, control condition) were tested with a paradigm of prepulse inhibition (PPI) of the acoustic startle reflex (ASR) to acquire baseline data for later tinnitus percept evaluation (cf. below). Second, ABR measurements of the same animals were performed to obtain individual audiograms under anesthesia and the far field responses of the auditory brainstem up to the inferior colliculus (IC). Following these baseline measurements, an acoustic trauma (2 kHz, 115 dB sound pressure level (SPL), 75 min) under deep anesthesia was used to induce a mild NIHL and was evaluated by ABR directly after the trauma. The animals were allowed one week of recovery during which a possible tinnitus percept could develop and become chronic; after that week the animals were either fed daily with EGb 761^®^ in agar (100 mg extract / kg body weight; EGb 761^®^ was provided by Dr. Willmar Schwabe Pharmaceutics (Karlsruhe, Germany) and diluted in 2% agar in water) via a feeding cannula over three weeks (group G, 11 animals), or they were fed over the same time with the same volume of agar only (group V, 9 animals). EGb 761^®^ is a dry extract from *Ginkgo biloba* leaves (35–67:1), extraction solvent: acetone 60% (w/w). The extract is adjusted to 22.0–27.0% ginkgo flavonoids calculated as ginkgo flavone glycosides and 5.0–7.0% terpene lactones consisting of 2.8–3.4% ginkgolides A, B, C, and 2.6–3.2% bilobalide and contains less than 5 ppm ginkgolic acids. During the three weeks (1^st^ to 3^rd^ week) and in the week after the end of the oral application (4^th^ week) a behavioral test for tinnitus (gap PPI of ASR, cf. below) followed by an ABR measurement was performed.

### Behavioral tinnitus test (gap PPI of ASR)

The setup for behavioral measurement has been described in detail earlier [29, 31]. Briefly, for behavioral testing, animals were placed in a transparent acrylic tube (length: 10 cm; inner diameter 4.3 cm). This tube was placed 10 cm in front of a speaker (Canton Plus X Series 2) onto a Honeywell FSG15N1A piezo force sensor (sensitivity 0.24 mV/gram; null shift at ±25°C is ±1mV; force range 0 to 1500 gram), assembled within an acoustic chamber (Industrial Acoustics, Niederkrüchten, Germany) on a TMC low-vibration table. The front end of the tube was closed with a stainless steel grate (wire mesh width 0.5 mm) allowing acoustic stimulation with no detectable distortion within the used stimulation range of 250 to 16000 Hz (signal to noise ratio at least 70 dB). Sound pressure level was controlled via a B&K Type 2610 measuring amplifier fed with a B&K Type 2669 preamplifier / B&K Type 4190 condensor microphone combination. Stimulus generation and data acquisition was controlled using custom-made Matlab 2008 programs (MathWorks, Natick, MA, USA; stimulation / recording sampling rate 20 kHz). For sound generation the frequency response function of the speaker was calibrated to produce an output spectrum that was flat within +/- 1 dB measured within the acrylic tube.

The PPI modulated ASR paradigm is used to assess the potential existence of a tinnitus percept [32, 33]. It consists of a 105 dB SPL pure tone startle stimulus of 1 kHz, 2 kHz, 4 kHz, 8 kHz or 16 kHz within a 50 dB SPL continuous white noise, a silent 15 ms gap within the noise 100 ms before the startle stimulus served in half of the trials as pre-pulse. Each frequency and gap-condition was repeated 15 times and each test was performed before and several times after the trauma (cf. Fig 1). Data obtained were checked by eye, independently by two experimenters via a custom made Matlab program. Trials in which the animals moved within 100 ms before the startle stimulus were discarded; in the remaining valid trials only peak-to-peak amplitudes of responses within the first 50 ms after startle stimulus onset were used for further analysis. The PPI of ASR was calculated [31]. The change in PPI relative to pre-trauma (in %, thereby taking into account absolute changes of PPI that may occur after NIHL) was tested against 0 (no change) for each frequency separately by t-tests (α = 0.025). Significant values for PPI change reflect impaired PPI and therefore indicate the development of a tinnitus percept in the tested frequency. The number of affected frequencies was counted for each animal and each measurement session. Note that out of the five tested tinnitus frequencies (1, 2, 4, 8 and 16 kHz) the animals in our experiments were positive for maximally three frequencies.

### ABR measurements

ABR data were obtained under deep anesthesia (mixture of ketamine, xylazine, NaCl and atropine at a mixing ratio of 9:1:8:2, initial dose: 0.3 ml subcutaneous; anesthesia was kept constant via a subcutaneous infusion with 0.2 to 0.3 ml/h.) via three subcutaneously placed thin silver wire electrodes, one for grounding at the back of the animals, one reference electrode at the forehead between the eyes and the ears and the measuring electrode infra-auricular overlying the bulla. Signals were recorded using a Plexon Multichannel Acquisition Processor (Plexon Inc., Dallas, TX, USA) after amplification by a JHM NeuroAmp 401 (J. Helbig Messtechnik, Mainaschaff, Germany; bandpass filter 400 to 2000 Hz, amplification 10000) and stored with a custom-made Matlab program (at 10 kHz sampling rate). Auditory stimuli were generated by a custom-made Matlab program and presented free field to one ear at a time via a frequency transfer function corrected speaker (SinusLive neo 25S, pro hifi, Kaltenkirchen, Germany) at 5 mm distance from the animal’s pinna while the contralateral ear was tamped with an ear plug; both ears were measured directly after each other without any predefined order. Stimuli presented were clicks (0.1 ms duration) and pure tones (4 ms duration including 1 ms cosine-squared rise and fall times) ranging from 0.5 to 16.0 kHz in half-octave steps with 120 repetitions each. Stimulation was pseudo-randomized using a fixed list of all combinations of stimulus frequencies and sound pressure levels (0 to 90 dB SPL in 5 dB steps). To obtain ABR-based audiograms the mean ABR wave amplitudes were compared to the mean amplitude 200 to 100 ms before the stimulus (baseline). Thresholds were defined automatically by a custom-made Matlab program at the highest attenuation at which the evoked amplitude raised over 2 standard deviations of the baseline; data were discarded at frequencies where this procedure was not possible, e.g., at low signal to noise ratios. For additional analysis the root mean square (RMS) value of the ABR signal was calculated from 1 to 5 ms after stimulus onset and single waves of the ABR responses representing different levels within the brainstem [34] were detected by a custom made Matlab program at 70 dB SPL for each stimulation frequency. According to our classification the identified waves were: wave I/II (nervus cochlearis and nucleus cochlearis), wave III (superior olivary complex/trapezoid body), wave IV (lateral lemniscus) and wave V (IC).

### Statistical analysis

All data were analyzed statistically using Statistica (StatSoft Inc., Tulsa, OK, USA). We analyzed ASR and ABR amplitude data by parametric tests, ABR wave latencies as well as the number of tinnitus frequencies were assessed by non-parametric tests as one cannot assume normal distribution for these parameters. In most parametric analyses we display the mean response amplitudes (± 95% confidence intervals, CI) by the interaction of two predictors derived from a 2-factorial ANOVA with or without one or both effects of the single factors. In the case of ABR wave amplitude analysis we performed a 3-factorial ANOVA. ABR wave latencies were assessed by non-parametric Kruskal-Wallis ANOVAs (post-hoc test: multiple comparison of mean ranks) and Mann-Whitney U-tests. The comparison of the number of tinnitus frequencies at different time points and between groups was assessed by Kolmogorov-Smirnov tests, corrected for multiple comparisons.

## Results

### Database

Data from a total of 20 male Mongolian gerbils were included in this study. Out of these, 11 were treated with *Ginkgo biloba* extract EGb 761^®^ (group G), while 9 received vehicle treatment (group V), according to the protocol presented in Fig 1.

### Effect of EGb 761^®^ treatment on noise induced hearing loss

**Fig 2:**
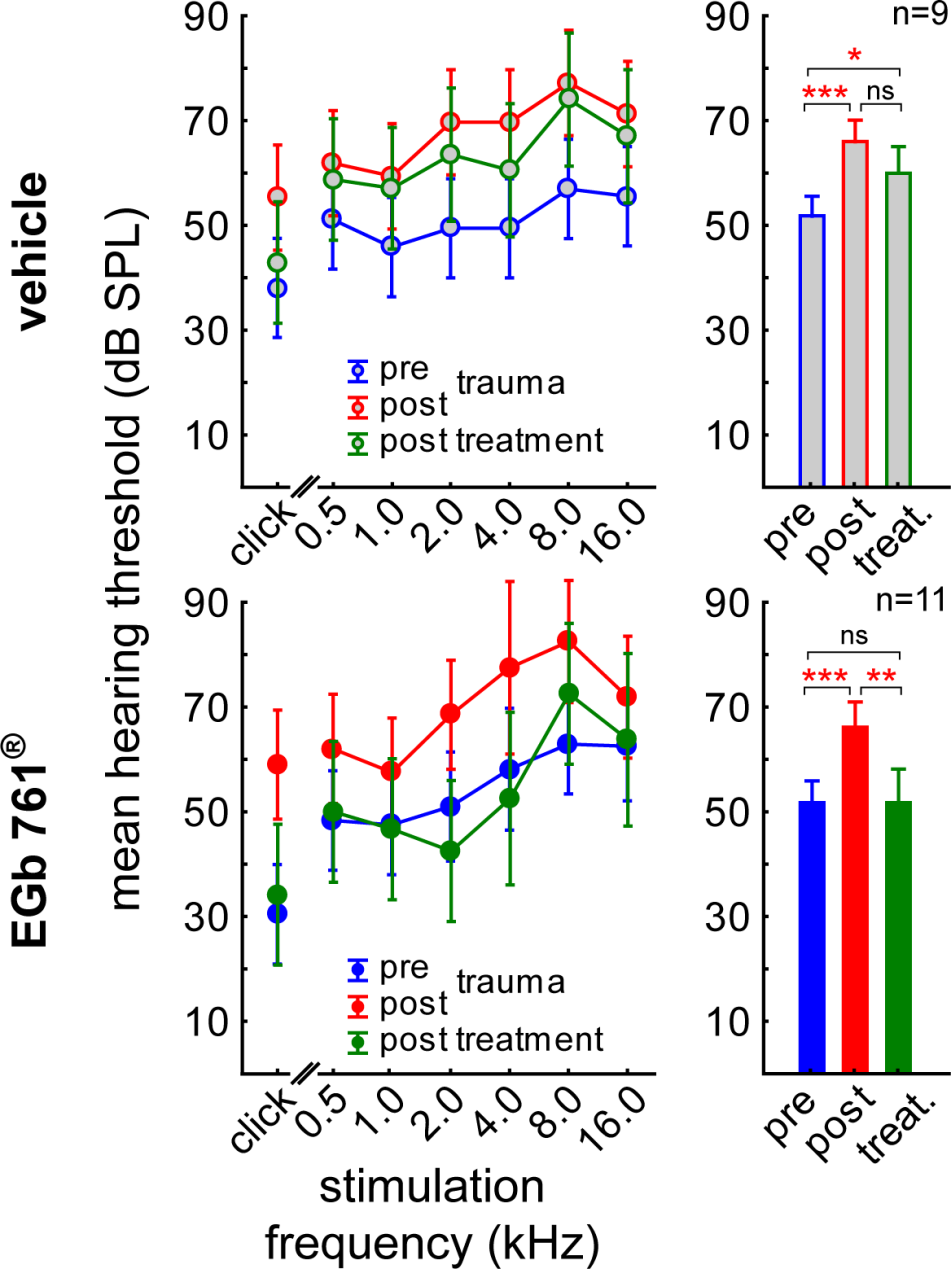
EGb 761 alleviates noise-induced increase in hearing thresholds. Left panels: Audiograms, right panels: mean thresholds. Results of the 2-factorial ANOVAs of mean hearing thresholds (± 95% CI) dependent on *time point* (blue: pre trauma, red: post trauma, green: post treatment) and *stimulation frequency*. Upper panels: Group V hearing thresholds interaction *time point x stimulation frequency* (F(12, 136) = 0.26, p = 0.99) and factor *time point* (F(2, 136) = 21.07, p<0.001). Lower panels: Group G hearing thresholds interaction *time point x stimulation frequency* (F(12, 66) = 0.71, p = 0.73) and factor *time point* (F(2, 66) = 18.97, p<0.001). Asterisks indicate the significant changes over time tested by Tukey post-hoc tests, ns not significant, * p<0.05, ** p<0.01, *** p<0.001.

Hearing thresholds ranging from 0.5 to 16 kHz were measured in all animals before noise trauma and did not differ significantly between groups V and G (mean ± standard deviation (SD): V: 53.46 ± 14.14 dB, G: 52.64 ± 15.13 dB; t-test: p = 0.71; cf. pre trauma audiograms (blue curves) and mean thresholds (blue bars) in Fig 2). Note that threshold estimates based on ABR measurements are relatively high compared to behavioral measurements [35], because the latter method is more sensitive than the former. Nevertheless, as we are mainly interested in relative threshold changes between pre and post trauma conditions which are independent of the method of threshold measurement [35], absolute thresholds are rather irrelevant for this study. Noise trauma induced a significant increase of mean threshold to V: 67.80 ± 15.13 dB; G: 66.21 ± 13.12 dB (Fig 2 red bars; Tukey post-hoc p<0.001 for V and G). This deterioration of hearing thresholds affected the whole frequency range tested, resulting in a parallel shift of the audiograms in both groups (2-factorial ANOVAs *pre vs*. *post trauma* x *frequency*, V: F(6, 201) = 1.27, p = 0.28; G: F(6, 256) = 1.09, p = 0.37). A first difference between groups was evident post treatment: Thresholds of treated animals recovered nearly completely after three weeks of EGb 761^®^, while those of the vehicle treated animals did not show further recovery. This is demonstrated by 2-factorial ANOVAs of the threshold dependent on the factors *time point* (pre trauma, post trauma and post treatment) and *frequency* for group V and G revealed a significant differences between those groups (Fig 2, green symbols and bars): While hearing thresholds in group G normalized back to levels not significantly different from pre trauma conditions (pre vs. post treatment, Tukey post-hoc test, p>0.05), no significant improvement was observed in group V (pre vs. post treatment, Tukey post-hoc test, p = 0.02 and post trauma vs. post treatment, Tukey post-hoc test, p>0.05).

### Effect of EGb 761^®^ treatment on tinnitus

**Fig 3:**
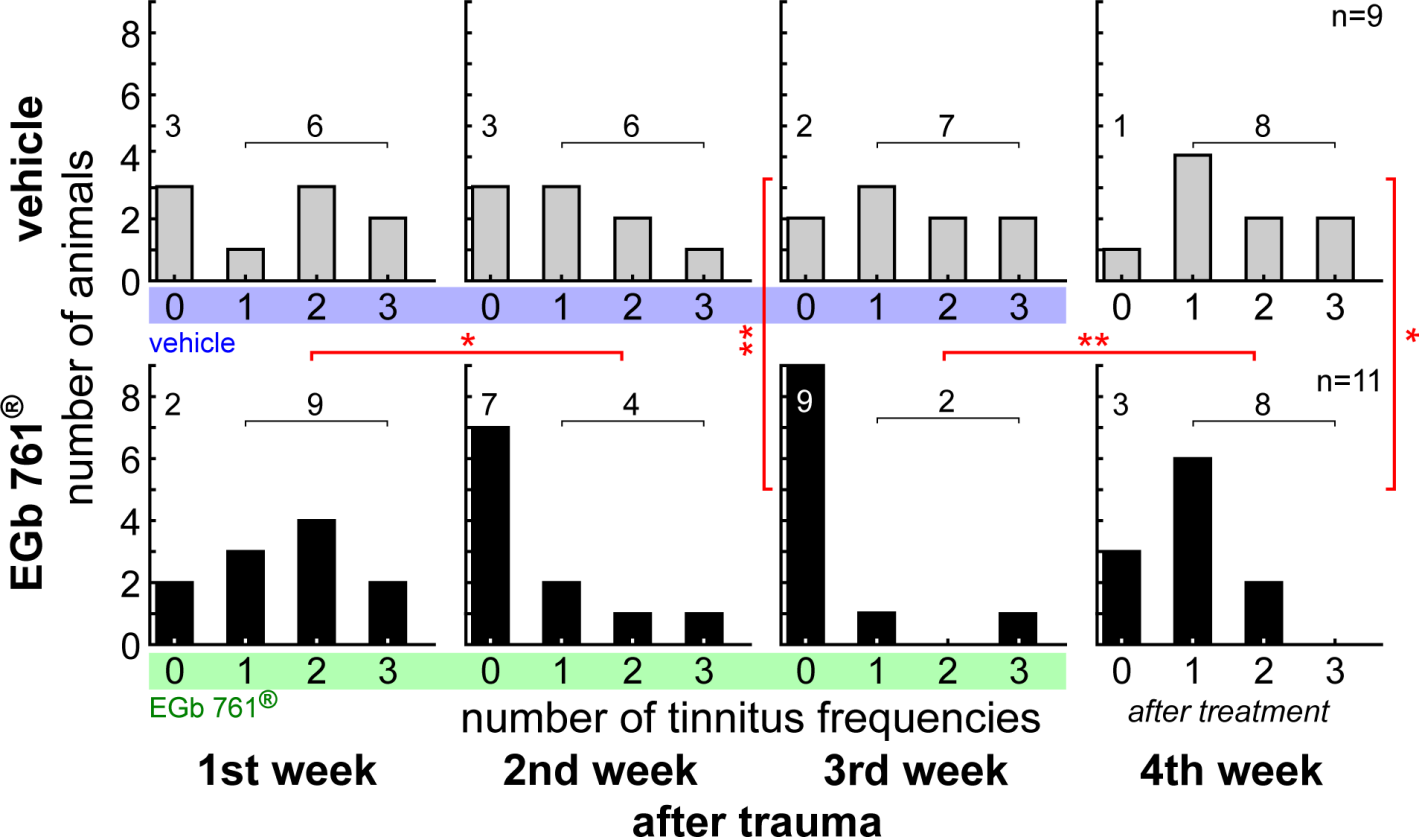
EGb 761 alleviates behavioral signs of noise-induced tinnitus perception. Given are the distributions of the number of perceived tinnitus frequencies as a function of the week relative to treatment onset. Numbers on the abscissae indicate how many frequencies were tested positive for a tinnitus percept in a given animal (cf. Methods), with zero referring to animals with no tinnitus, and one to three referring to the number of affected frequencies that may be taken as a measure for tinnitus severeness. The ordinates give numbers of animals affected accordingly. Note that the distributions of the two groups were tested at each time point against each other as well as within one group between different time points. Asterisks indicate the significant changes over time or the differences between the groups tested by Kolmogorov-Smirnov tests corrected for multiple comparisons, * p<0.05, ** p<0.01.

Treatment with EGb 761^®^ did not only improve hearing thresholds in NIHL, it also reduced the behavioral signs of tinnitus, as displayed in Fig 3. At the beginning of treatment (left panels in Fig 3), 3 animals in group V showed no behavioral signs of tinnitus, while 6 animals showed different degrees (one to three) of tinnitus severity. In group G, 2 animals had no tinnitus at treatment onset, while 9 show tinnitus in our behavioral paradigm. There was no significant difference between groups in the distribution of the number of affected frequencies before treatment (Kolmogorov-Smirnov, p>0.05).

During the second week of treatment, this situation did not change in group V, whereas there was a significant improvement in group G where now 7 animals were free of behavioral signs of tinnitus while only 4 where still positive for the phantom percept (G 1^st^ vs. 2^nd^ week, Kolmogorov-Smirnov, p = 0.04). In the third week of treatment the situation in group G was even further improved with only 2 out of 11 animals still being positive for tinnitus (G 1^st^ vs. 3^rd^ week, Kolmogorov-Smirnov, p<0.009). By contrast, the situation in group V did not change significantly (V 1^st^ vs 3^rd^ and 2^nd^ vs 3^rd^, Kolmogorov-Smirnov, p>0.05 each) but the difference between both groups in the third week became significant (V vs. G 3^rd^ week, Kolmogorov-Smirnov, p = 0.003).

After the end of treatment, group V did still show the same distribution of number of tinnitus frequencies as at the beginning of treatment (V 1^st^ vs. 4^th^ week, Kolmogorov-Smirnov, p = 0.95). After discontinuation of EGb 761^®^ treatment tinnitus reappeared (within 3 to 4 days) in 6 animals of group G (G 3^rd^ vs. 4^th^ week, Kolmogorov-Smirnov, p = 0.01).

### Effect of EGb 761^®^ treatment on auditory brainstem responses

**Fig 4:**
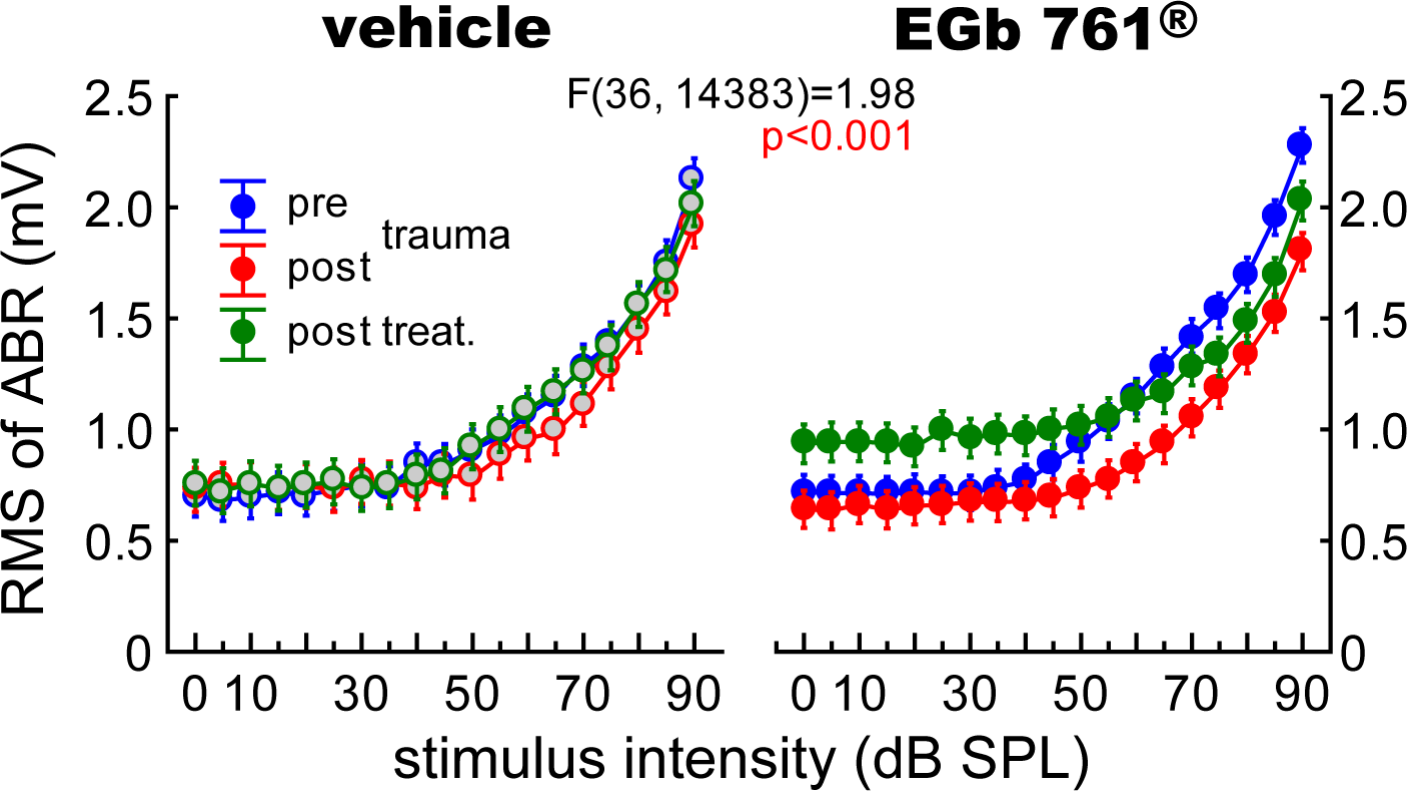
The ABR suggests EGb 761-induced changes in central auditory processing. Root mean square (RMS) of ABR amplitudes (with an amplification factor of 10.000) of vehicle and EGb 761^®^ treated animals. 3-factorial ANOVA of the mean RMS values across all frequencies (± 95% CI) dependent on *group*, *time point* and *stimulus intensities*. Statistics given refer to the interaction of the three factors.

**Fig 5:**
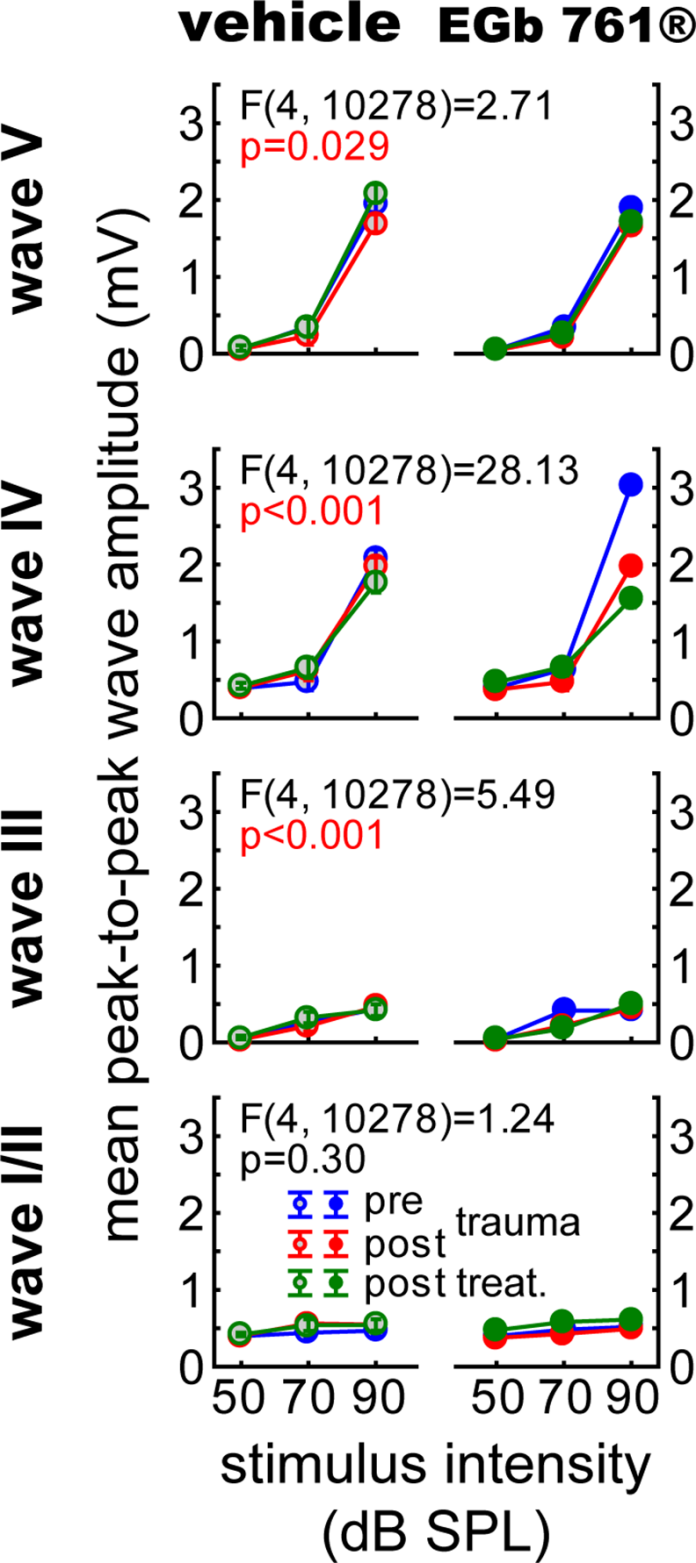
Detailed analysis of ABR waves further support EGb 761- induced changes in central auditory processing. Mean peak-to-peak amplitudes (with an amplification factor of 10.000) of single ABR waves of vehicle and EGb 761^®^ treated animals. 3-factorial ANOVAs for each wave complex (I/II, III, IV and V) of the mean amplitude values (± 95% CI) over all frequencies dependent on *group*, *time point* and *stimulus intensities*. The panels depict the interactions of the three factors for the different waves.

**Fig 6:**
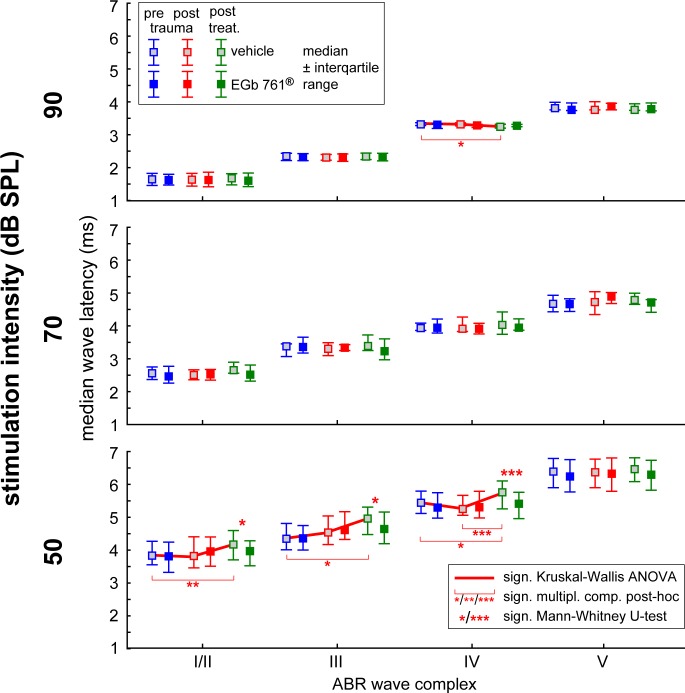
EGb 761 alters median ABR wave latencies. Median latencies (± interquartile interval) are given for three different stimulus intensities (90, 70, 50 dB SPL) and three different time points (before trauma, immediately after trauma, and after treatment). Red lines indicate significant Kruskal-Wallis ANOVAs over the three *time points* within one group and wave data, Asterisks indicate levels of significance in Mann-Whitney U-tests between the groups at one time point. * p<0.05, *** p<0.001.

EGb 761^®^ treatment induced changes in central auditory processing as assessed by ABR measurements that were not seen in vehicle treated animals as depicted in Figs 4–6.

First, both animal groups showed an initial drop in auditory brainstem activity after trauma (Fig 4, blue to red symbols). After treatment (Fig 4, green symbols) the vehicle group remained on that response level while the EGb 761^®^ treated animals showed intensity dependent changes in auditory brainstem activity with an increase at lower intensities and a stabilization of reduced activity at higher intensities. In detail, the interaction of the 3-factorial ANOVA of the mean RMS values of ABR across all frequencies are given in Fig 4 as a function of *stimulation intensity*, *time point* and *group*. Generally, in both *groups* noise trauma led to a decrease of ABR amplitudes (Tukey post-hoc tests, V pre: 1.08 ± 0.73 mV vs post: 0.88 ± 0.57 mV, p = 0.002, G pre: 1.25 ± 0.67 mV vs post: 0.97 ± 0.96 mV, p<0.001). Analyzing the pre trauma and post treatment data in greater detail we find that in group G the EGb 761^®^ treatment did not change ABR amplitudes back to normal but resulted in an increase of RMS values at low intensity (up to 50 dB SPL) to values above pre trauma conditions (mean RMS pre: 0.75 ± 0.26 mV, mean RMS post treatment: 0.97 ± 0.45 mV, t-test, p<0.001), while for higher stimulus intensities RMS values remained below pre trauma values (mean RMS pre: 1.54 ± 0.90 mV, mean RMS post treatment: 1.39 ± 0.78 mV, t-test, p<0.001). No such effect was seen in group V, where we only saw the general amplitude decrease in both intensity ranges (up to 50 dB: mean RMS pre: 0.74 ± 0.33 mV, mean RMS post treatment: 0.71 ± 0.24 mV, t-test, p = 0.005; above 50 dB: mean RMS pre: 1.94 ± 0.95 mV, mean RMS post treatment: 1.40 ± 0.79 mV, t-test, p = 0.002).

Second, Figs 5 and 6 present a more fine-grained analysis of ABR measurements. When analyzing single wave amplitudes, representing the different brainstem nuclei of the auditory pathway, we found a stabilizing effect of EGb 761^®^ above the level of the DCN (waves III to V; cf. Fig 5) especially in mid-intensity stimuli of 70 dB SPL. The same was true for latencies to low intensity stimulation at auditory brainstem levels below IC (waves I to IV; Fig 6). In Fig 5, these changes in mean peak-to-peak amplitudes of ABR waves across all frequencies and for three stimulus intensities in the course of experiments of both groups are given (for mean values with standard deviation please refer to Table 1). In general, the development of peak-to-peak amplitudes over time and intensities in group V (Fig 5, left column) was significantly different from that in group G (Fig 5, right column) in waves III to V but not in the earliest waves I/II. This is especially prominent, first, in wave IV, where in group V only at the 90 dB stimulation the amplitudes are significantly reduced in the post treatment condition (green) compared to the pre trauma condition (blue; Tukey post-hoc test, p<0.001) and, second, in wave V of that group where at 90 dB stimulation only the amplitudes in the post treatment condition (green) are significantly increased compared to post trauma condition (red; Tukey post-hoc test, p<0.001). In contrast, in group G the amplitudes of wave IV showed at the 90 dB stimulation steadily decreases over time (pre vs. post trauma, post trauma vs. post treatment, pre vs. post treatment, Tukey post-hoc tests, p<0.001 each) and a significant increase of amplitude at 50 dB SPL when comparing post trauma vs. post treatment condition (Tukey post-hoc test, p<0.001). In wave V of group G a significant decrease of amplitude can only be found at 90 dB pre vs. post trauma condition (Tukey post-hoc test, p<0.001). Further differences between the two groups are found in wave III, where we see no significant effects on the amplitudes of group V at any intensity or point in time, but in group G a significant decrease of amplitudes at 70 dB post trauma and post treatment relative to the pre trauma condition (Tukey post-hoc tests, p<0.001 each). In wave I/II again no changes in group V could be found, but in group G a significant increase of amplitudes at 50 and 70 dB post treatment relative to post trauma (Tukey post-hoc tests, p<0.001 each) arose, the 50 dB increase post treatment is also significant against the pre trauma amplitude (Tukey post-hoc test, p<0.001).

**Table 1:** Mean response amplitudes of ABR waves (mV) at an amplification of 10.000:.

animal group	intensity (dB SPL)	ABR wave	pre trauma (mean ± SD)	post trauma (mean ± SD)	post treatment (mean ± SD)
vehicle	50	I/II	0.49 ± 0.04	0.42 ± 0.04	0.36 ± 0.04
		III	0.13 ± 0.06	0.10 ± 0.03	0.19 ± 0.06
		IV	0.50 ± 0.04	0.41 ± 0.03	0.57 ± 0.05
		V	0.16 ± 0.07	0.16 ± 0.06	0.16 ± 0.05
	70	I/II	0.46 ± 0.05	0.57 ± 0.06	0.55 ± 0.05
		III	0.26 ± 0.08	0.23 ± 0.08	0.31 ± 0.09
		IV	0.47 ± 0.05	0.63 ± 0.07	0.67 ± 0.06
		V	0.39 ± 0.12	0.26 ± 0.08	0.36 ± 0.11
	90	I/II	0.45 ± 0.04	0.57 ± 0.05	0.55 ± 0.05
		III	0.43 ± 0.04	0.49 ± 0.05	0.43 ± 0.03
		IV	2.15 ± 0.18	2.08 ± 0.17	1.75 ± 0.13
		V	2.16 ± 0.19	1.73 ± 0.20	2.15 ± 0.19
EGb 761^®^	50	I/II	0.49 ± 0.06	0.47 ± 0.04	0.56 ± 0.04
		III	0.14 ± 0.05	0.11 ± 0.04	0.12 ± 0.04
		IV	0.51 ± 0.06	0.49 ± 0.04	0.56 ± 0.04
		V	0.13 ± 0.04	0.10 ± 0.03	0.15 ± 0.04
	70	I/II	0.46 ± 0.03	0.44 ± 0.03	0.58 ± 0.06
		III	0.45 ± 0.18	0.23 ± 0.10	0.19 ± 0.05
		IV	0.66 ± 0.08	0.46 ± 0.06	0.67 ± 0.07
		V	0.36 ± 0.11	0.23 ± 0.09	0.28 ± 0.08
	90	I/II	0.50 ± 0.04	0.50 ± 0.04	0.61 ± 0.05
		III	0.40 ± 0.03	0.45 ± 0.04	0.51 ± 0.04
		IV	3.14 ± 0.31	2.07 ± 0.22	1.58 ± 0.12
		V	1.92 ± 0.11	1.73 ± 0.20	1.67 ± 0.16

The detailed analysis of ABR wave latencies is given in Fig 6. Median latencies across all stimulus frequencies are shown for three different intensities. Interestingly, significant changes in wave latency in the course of experiments were only seen in group V (Kruskal-Wallis ANOVAs, p<0.001 each), pointing to a stabilizing effect of EGb 761^®^ treatment: Wave latencies in group V were significantly increased at 50 dB SPL for waves I/II (multiple comparison of means (MCM), p = 0.01), III (MCM, p = 0.004), and IV (MCM, p<0.001) between post trauma and post treatment conditions, indicating a late effect of noise trauma on ABR wave latencies. This latency increase of group V results also in the significant differences between these values and the latencies of group G waves (Mann-Whitney U-tests, p values between p = 0.03 and p<0.001, cf. Fig 6 left panel). By contrast, wave IV latencies at 90 dB SPL were significantly decreased in group V (MCM, p = 0.01) relative to the pre trauma condition but did not differ from group G latency (Mann-Whitney U-test, p = 0.13). No significant changes of ABR wave latency were observed in group G in any condition.

## Discussion

In this study, we found that EGb 761^®^ was beneficial for the treatment of existing NIHL and tinnitus: three weeks of EGb 761^®^ treatment led to a persistent recovery of auditory thresholds back to pre-trauma conditions. In addition we saw an amelioration of behavioral signs of tinnitus–even though we are aware that the method we used in our animal model to assess tinnitus provides an indication rather than a proof for the existence of tinnitus (cf. below): 7 of 9 animals initially positive for behavioral signs of tinnitus presented themselves free of this phantom percept after three weeks of treatment. Tinnitus seemed to reappear after EGb 761^®^ treatment was discontinued.

### Effect of Ginkgo extracts on tinnitus in humans

The question whether *Ginkgo* extracts are beneficial for human tinnitus sufferers is still discussed controversial. Eight clinical studies including a total of 1199 patients, using the extract EGb 761^®^ demonstrated a positive effect in favor of active treatment [36]. That systematic review included studies with patients suffering from long-lasting tinnitus over several years, as well as tinnitus as a symptom in patients with dementia. EGb 761^®^ is an approved medication for tinnitus in Germany and many other countries world-wide. However, such a positive effect could not be demonstrated using other Ginkgo extracts [37]. Controlled animal studies like the one presented here may help to elucidate the mechanism involved in Ginkgo extract based tinnitus treatment.

### Reliability of animal models of tinnitus

To assess tinnitus in our animal model we use the gap-startle paradigm introduced by Turner and colleagues [27, 29, 30, 38]. It is based on the idea that detection of a silent period (gap) in a background noise is impaired if animals perceive their own tinnitus during the gap. The reliability of this method for the detection of tinnitus has been discussed controversial [39, 40], mainly because it relies on PPI of a reflex, the ASR, that in mammals relies on the neural circuitry of the pons [41] and does not involve cortical processing, whereas tinnitus is considered to emerge from neuronal activity within the auditory cortex. Nevertheless, it has been shown repeatedly that gap detection relies on the auditory cortex (e.g., [42]) so that a cortical, top-down influence on the PPI of the ASR is obvious if a gap is used as pre-pulse.

In contrast to other animal models of tinnitus, the gap-startle model is the only one that does not rely on some conditioning paradigm where animals are trained to respond to a potential tinnitus percept in a certain way. As we are interested not only in the existence of a tinnitus percept in our animals per se but also in the neuronal plasticity that leads to tinnitus, all animal models that involve conditioning paradigms are inappropriate for such purposes as they trigger learning-induced neuronal plasticity that cannot be disentangled from tinnitus related plasticity. On the other hand, we were able to show that in our model the neuronal plasticity that can be monitored in auditory brainstem as well as auditory cortex is different between animals that develop behavioral signs of tinnitus in the gap-startle paradigm compared to those that do not (cf. [29, 30]), thereby supporting the interpretation that animals being positive in the behavioral test indeed perceive tinnitus.

In the present study, we used the identical animal model for tinnitus as in our previous studies to test for the effects of EGb 761^®^ on this condition. Nevertheless, it may not be excluded completely that the beneficial effect of the treatment on the behavioral signs of tinnitus that we observed does not reflect alleviation of tinnitus: As EGb 761^®^ has been shown to also reduce stress levels and improve mood (e.g., [43, 44]) which may both have impact on tinnitus severity [45, 46], such effects may also underlie the changed outcome of our behavioral test for tinnitus. The reappearance of the tinnitus percept after discontinuation of treatment can also be due to a too short application of EGb 761^®^, resulting in only temporary relief or may be an indication for a lack of power to reverse maladaptive cortical plasticity (but cf. [27]). Nevertheless, the ABR data presented in Figs 4–6 demonstrate subtle changes in sound processing within the auditory system following EGb 761^®^ treatment (cf. below). As stress levels and mood mainly depend on processing within the limbic system, it seems likely that the treatment had at least some effect on auditory perception, including tinnitus.

There are a number of other reports of pharmacological effects on the perception of tinnitus in animal models. For example, Guitton and Dudai [47] were able to show that an intervention by an intra-cochlear injection of the NMDA antagonist "ifenprodil" within the critical time window of four days after trauma could prevent the long term development of a tinnitus percept in 50% of the used rats. Sendowsk and colleagues [48] were able to achieve nearly complete restoration of hearing thresholds by intra-cochlear injections of methylprednisolone over 7 days around the critical period and by that prevention of tinnitus development. And Zheng and coworkers [49] temporarily reduced the chronic tinnitus percept of rats by sub-cutaneous injections of L-baclofen until washout for several hours.

### Neuronal plasticity triggered by EGb 761^®^

The data presented in Figs 4–6 show changes in central auditory processing induced by noise trauma (blue to red) and subsequently by EGb 761^®^ treatment (red to green); also cf. S1 File. Interestingly, the EGb 761^®^ treatment does not restore the pre-trauma conditions. Therefore, the beneficial effects of the treatment on NIHL (Fig 2) and tinnitus (Fig 3) observed here obviously do not reflect a reversal of noise trauma induced neurophysiological maladaptations that led to tinnitus, but rather point to additional, compensatory plasticity triggered by the treatment. In particular, overall ABR amplitudes to auditory stimuli of low intensity are increased after EGb 761^®^ treatment which may reflect the improvement of hearing thresholds (Fig 4). By contrast, ABR amplitudes to auditory stimuli of high intensity are decreased after EGb 761^®^ treatment (Fig 4), possibly reflecting a global inhibitory mechanism that counteracts the development of tinnitus, as has been proposed earlier [29, 30]. This mechanism seems to work on the higher brainstem nuclei like the IC, as we see strongest effects in the corresponding waves (Fig 5). With respect to response latencies (Fig 6) we saw a lack of latency change under EGb 761^®^ treatment compared to the vehicle group. In other words, the extract seems to stabilize the response latencies which otherwise show (mal-)adaptive plasticity post trauma. As this effect is most prominent in responses to near threshold signals Fig 6, bottom panel) it may reflect the recovery of thresholds in the treated group. In general, EGb 761^®^ seems to stabilize auditory responses at some medium level as has been reported previously [27].

The EGb 761^®^ dosage that we used in this study (100 mg/kg) is roughly comparable to the dosing in human: Taking into account the about 10-fold higher metabolic rate of gerbils compared to humans, the effective dose used in our animal studies compares to about 700 mg daily dose in humans, which is about 3 times as the highest actually recommended daily dose for humans (240 mg/d). In this context it is worth noting that dosages up to 480 mg/d have been reported to be well tolerated in humans [44].

In summary, the data presented in this study suggest that the beneficial effect of high dosage EGb 761^®^ treatment observed in NIHL and tinnitus in our animal model may involve modification of central auditory processing. Our result provides additional information on the processes that may be involved in the alleviation of symptoms in tinnitus by treatment with EGb 761.

## Supporting Information

S1 File Statistica files for comprehension of our data analysis. (ZIP)
